# Chronic stress, neuroinflammation, and depression: an overview of pathophysiological mechanisms and emerging anti-inflammatories

**DOI:** 10.3389/fpsyt.2023.1130989

**Published:** 2023-05-11

**Authors:** Sameer Hassamal

**Affiliations:** ^1^California University of Sciences and Medicine, Colton, CA, United States; ^2^Clinicaltriallink, Los Angeles, CA, United States; ^3^California Neuropsychiatric Institute, Ontario, CA, United States

**Keywords:** depression, stress neuroinflammation, cortisol, HPA axis, neuroplasticity Glutamate, GABA

## Abstract

In a subset of patients, chronic exposure to stress is an etiological risk factor for neuroinflammation and depression. Neuroinflammation affects up to 27% of patients with MDD and is associated with a more severe, chronic, and treatment-resistant trajectory. Inflammation is not unique to depression and has transdiagnostic effects suggesting a shared etiological risk factor underlying psychopathologies and metabolic disorders. Research supports an association but not necessarily a causation with depression. Putative mechanisms link chronic stress to dysregulation of the HPA axis and immune cell glucocorticoid resistance resulting in hyperactivation of the peripheral immune system. The chronic extracellular release of DAMPs and immune cell DAMP-PRR signaling creates a feed forward loop that accelerates peripheral and central inflammation. Higher plasma levels of inflammatory cytokines, most consistently interleukin IL-1β, IL-6, and TNF-α, are correlated with greater depressive symptomatology. Cytokines sensitize the HPA axis, disrupt the negative feedback loop, and further propagate inflammatory reactions. Peripheral inflammation exacerbates central inflammation (neuroinflammation) through several mechanisms including disruption of the blood–brain barrier, immune cellular trafficking, and activation of glial cells. Activated glial cells release cytokines, chemokines, and reactive oxygen and nitrogen species into the extra-synaptic space dysregulating neurotransmitter systems, imbalancing the excitatory to inhibitory ratio, and disrupting neural circuitry plasticity and adaptation. In particular, microglial activation and toxicity plays a central role in the pathophysiology of neuroinflammation. Magnetic resonance imaging (MRI) studies most consistently show reduced hippocampal volumes. Neural circuitry dysfunction such as hypoactivation between the ventral striatum and the ventromedial prefrontal cortex underlies the melancholic phenotype of depression. Chronic administration of monoamine-based antidepressants counters the inflammatory response, but with a delayed therapeutic onset. Therapeutics targeting cell mediated immunity, generalized and specific inflammatory signaling pathways, and nitro-oxidative stress have enormous potential to advance the treatment landscape. Future clinical trials will need to include immune system perturbations as biomarker outcome measures to facilitate novel antidepressant development. In this overview, we explore the inflammatory correlates of depression and elucidate pathomechanisms to facilitate the development of novel biomarkers and therapeutics.

## 1. Introduction

Depression is quickly becoming a public health crisis despite advances in diagnostics and therapeutics. At the height of the COVID-19 pandemic, the prevalence of depression tripled from 10 to 30% in the U.S. (1). The sharp rise in depression has been attributed to chronic stress stemming from social isolation and global economic uncertainty amid the largest land war in Europe since World War Two (2). Moreover, COVID-19 infection itself triggers an inflammatory cascade leaving the population primed for the development of neuroinflammation by an interplay between physiologic and psychological stress. Persistent stress exposure particularly among susceptible individuals with adverse childhood experiences induces chronic low-grade inflammation and increases the risk for emotional disturbances. Several lines of evidence support a link between chronic stress, low-grade inflammation, and depressive symptoms (3). Thus far, the evidence has been observational in nature and does not necessarily establish causality.

Stress induced hyperactivation of immunological processes represents a distinct pathway underlying depression. Innate and adaptive immune responses compromise the integrity of the blood brain barrier propagating bidirectional inflammatory signals between the periphery and central nervous system (4). Neuroinflammation, defined as activation of the central innate immune system, results in a depressive phenotype manifested with severe symptomatology and greater morbidity and mortality (5). A large body of evidence supports a positive association between higher serum and cerebrospinal fluid (CSF) concentrations of inflammatory cytokines, depression severity, and treatment resistance (6). Normalizing immune signaling, cytokine expression, and the nitro-oxidative balance reduces the severity of depression (7). The precise mechanistic pathways linking inflammation and depression remains to be elucidated, however, a plausible explanation is the disruption of neurotransmitters and neural networks distinct to depression.

**Figure 1:**
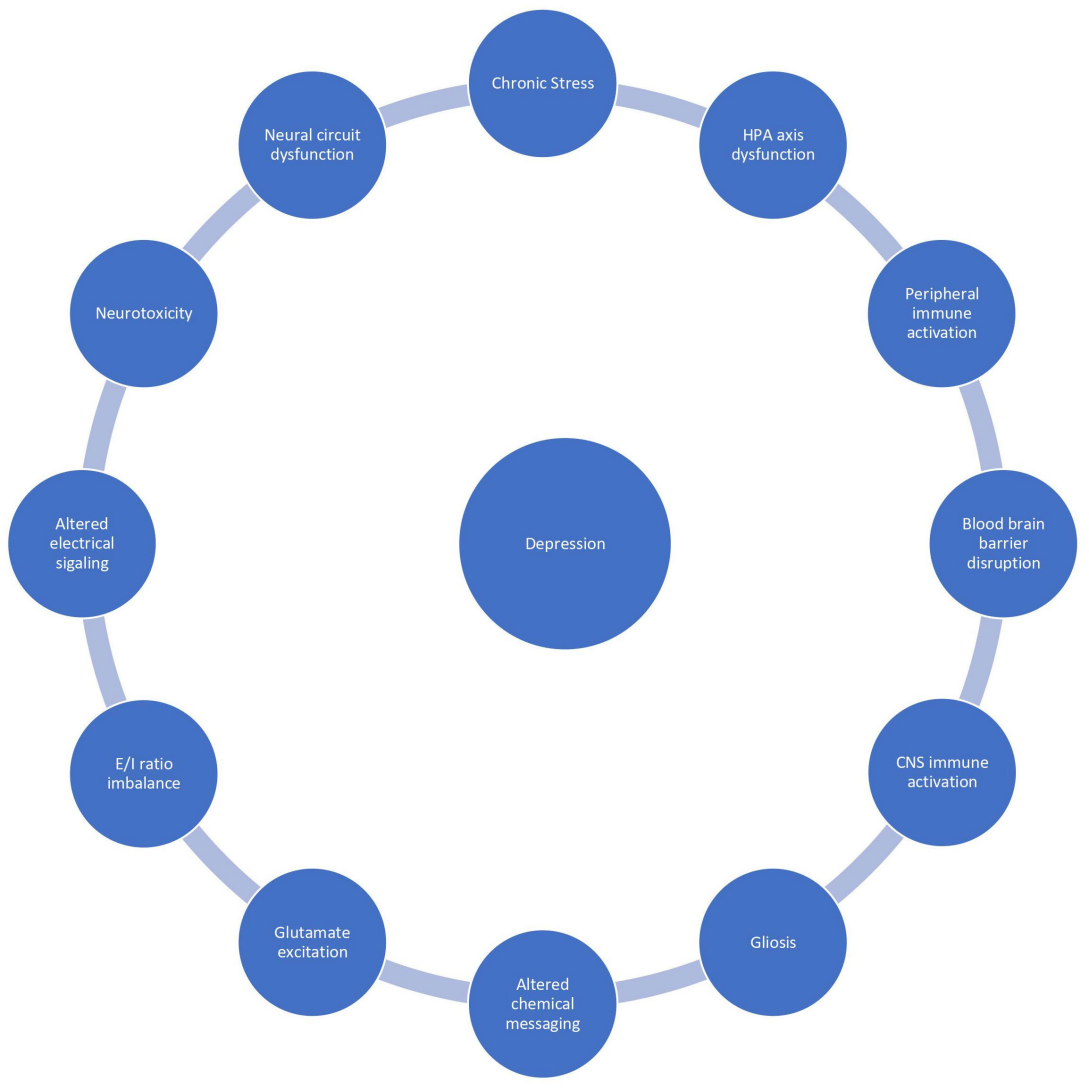
The vicious reciprocal relationship between chronic stress, inflammation, and depression.

Neuroinflammation affects 1 in 4 patients with depression and is associated with a poor prognosis, treatment resistance, and a reduced health related quality of life. There is no unifying diagnostic criteria for depression associated with inflammation, however a peripheral C-reactive protein (CRP) level exceeding 3 mg/L is commonly used as a marker of subtyping inflammation (8). Peripheral CRP levels strongly correlate with CSF CRP levels and are a reliable marker of neuroinflammation (9). Peripheral CRP levels exceeding 3 mg/l are associated with a specific depressive phenotype resembling “sickness behavior”: anhedonia, apathy, decreased appetite, fatigue, sleepiness, pain, suicidality, and cognitive impairments (5). These symptoms are present across disorders suggesting neuroinflammation underlies the shared variance between neuropsychiatric disorders. Exploring the inflammatory correlates of depression will elucidate pathomechanisms and facilitate the development of novel biomarkers and therapeutics. In this overview, we summarize the literature on putative mechanisms underlying stress dysregulation of the immune system, neuroinflammation, and depression (Figure 1).

## 2. The link between stress, inflammation, and depression

Preclinical data laid the foundation for understanding the relationship between stress, inflammation, and depression. Various forms of stress exposure (chronic mild stress, learned helplessness, repeated social defeat stress) induce a melancholic phenotype of depression, increase insulin insensitivity and levels of pro-inflammatory cytokines. Peripheral levels of IL-1β, IL-6, and TNF-α are consistently upregulated in susceptible animal models (10). Hodes and colleagues found that peripheral levels of IL-6 were 27 times higher in susceptible compared to resilient mice after repeated social defeat stress. Administering an IL-6 monoclonal antibody 5 min prior to repeated social defeat stress prevented the development of social avoidance suggesting that IL-6 contributes to stress-induced vulnerability (11). In a similar study, stress susceptible mice exhibited depressive-like behaviors that were interrelated with adrenocortical activation, increased IL-1 β levels in the hippocampus, and reduced hippocampal neurogenesis. Removing the adrenal glands abolished the depressive behaviors. In contrast, administering corticosterone amplified the depressive behaviors and reduced neurogenesis in mice with and without the IL-1R. The data suggests that adrenocortical activation mediates the relationship between IL-1 and stress-induced depression (12). Additional preclinical studies have established a relationship between cortisol dysregulation and depression. Lu and colleagues found that deletion of the astrocytic glucocorticoid receptor NR3C1 gene induced social avoidance and downregulated ATP release via the PI3K-AKT signaling pathway. The PI3K-AKT signaling pathway regulates physiological processes important for cell survival (13). Translational research has demonstrated that differential epigenetic NR3C1 gene hypermethylation patterns in individuals with a history of adverse childhood experiences contributes to a stress sensitive HPA axis response (14). Overall, the data suggest that aberrant glucocorticoid signaling mediates the relationship between the stress response, inflammation, and depression (15).

## 3. Hypothalamic–pituitary–adrenal axis

The initial mechanism linking chronic stress, inflammation, and depression can be explained by the dysregulation of the HPA axis and activation of immune response patterns. Typically, under normal conditions, cortisol decreases inflammation. However, persistent stress overstimulates the HPA axis causing an excess release of cortisol. Consequently, a dysregulation of the glucocorticoid negative feedback loop occurs due to glucocorticoid receptor resistance resulting in immune cell pro-inflammatory cytokine release (16). The dexamethasone suppression test (DST) is commonly used to assess HPA axis functioning. Low dose dexamethasone suppresses the release of cortisol and failure to do so is diagnostic of HPA axis hyperactivity and hypercortisolism (17). Dexamethasone fails to suppress adrenocorticotrophic hormone (ACTH) and cortisol in a subset of patients with melancholic depression suggesting underlying HPA axis dysfunction. Higher serum levels of cortisol and adrenocorticotrophic hormone (ACTH) have been reported in patients with severe and melancholic depression. In addition, urinary free cortisol levels are approximately two times higher in depressed patients compared to non-depressed patients (18). Overall, the sensitivity of the DST in diagnosing very severe cases of melancholic depression is approximately 70% (19).

**Figure 2:**
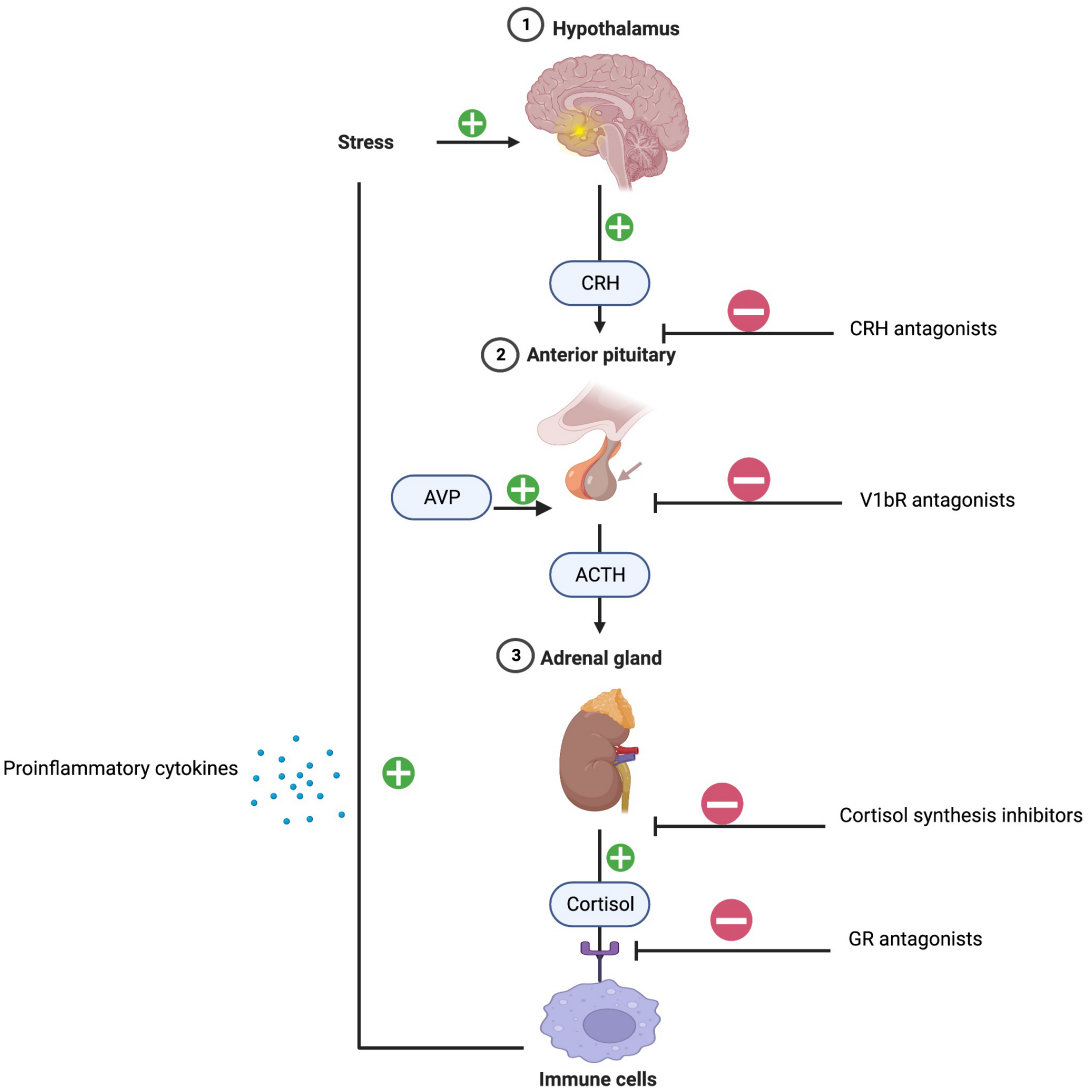
HPA-axis and inflammation. Created with BioRender.com.

Targeting HPA axis overactivity is a focus of antidepressant drug development. Pharmacotherapies antagonizing the GR and inhibiting cortisol synthesis have been developed, however, their efficacy has been stymied by phenotypic and genotypic diagnostic heterogeneities (20). More recent work has focused on arginine-vasopressin (AVP) and the vasopressin v1b receptor (V1BR) as primary factors contributing to HPA axis dysregulation. AVP-V1BR binding stimulates the pituitary release of ACTH and the secretion of cortisol. Multiple studies have reported that AVP levels are increased in the plasma and brain nuclei of depressed patients, indicating that overactivity of AVP-V1bR dysregulates the HPA axis (21). Thus far, the antidepressant effects of V1bR antagonists in small clinical trials are mixed but show efficacy signals in patients with overactive HPA activity (22) (Figure 2).

## 4. Damage-associated molecular patterns

Stress induces cells to release damage-associated molecular patterns (DAMPs), endogenous danger signals, into the extracellular space. DAMPs bind to pattern-recognition receptors (PRRs) on immune cells and activate the innate immune response. Several DAMPs have been implicated in depression, including high mobility group box-1 (HMGB-1), extracellular ATP, purine bases and metabolites, heat shock proteins (HSPs), S100 proteins, and galectin- 3 (Gal-3) (23). Studies have focused on S100 proteins, HSPs, and Gal-3 because of their pathogenic immunoregulatory roles in comorbid inflammatory diseases (24).

### 4.1. S100B

The S100B protein is highly expressed in neuronal cells, especially astrocytes. S100 B can either trigger trophic or inflammatory responses depending on the extracellular concentration. S100 B induces neurogenesis at nanomolar concentrations and inflammation at micromolar concentrations (25). Wang and colleagues reported that 43 days of chronic unpredictable mild stress increased hippocampal S100b expression in susceptible rats. Venlafaxine reduced hippocampal S100b expression and reversed depressive-like behavior suggesting that high levels of S100b predict the risk for depression and antidepressant response (26). In addition, S100B has been linked to suicide, and Dogan and colleagues found that CSF levels of S100B were higher in completed suicide autopsy cases compared to controls (27). Several clinical studies reported that serum levels of S100B are correlated with depression severity and recurrence. A study examining soldiers during intense combat training found that higher serum levels of S100 B correlated with greater distress levels and pro-inflammatory cytokines. The clinical data clearly support an association but not necessarily a causal link between S100 B, inflammation, and depression (28).

S100B is linked to comorbid inflammatory conditions, such as diabetes and metabolic syndrome. Adipose tissue contains S100B, which predisposes obese patients to depression. Diabetes, hypertension, high cholesterol, and metabolic syndrome (29). In a nested case–control study, patients with metabolic syndrome were 1.5 times more likely to use antidepressants compared to patients without metabolic syndrome (30). Higher levels of S100B are correlated with lower levels of serum-insulin one hour after an oral glucose tolerance test, suggesting that insulin tightly regulates S100B secretion from adipocytes (31). A 12-week randomized, placebo-controlled study found that pioglitazone, an insulin-sensitizing drug, improved glucose control and reduced chronic depression in patients with type II diabetes (28). Furthermore, emerging data supports that glucagon-like peptide-1 (GLP-1) agonists, such as liraglutide, have pro-cognitive, neuroprotective, and antidepressant effects (32). Overall, regulating S100B and glucose metabolism is likely to alleviate depression in patients with metabolic disorders.

### 4.2. HSPs

HSP’s are a molecular class of chaperones that help client proteins fold correctly and are pivotal to maintaining cellular integrity during stress. HSPs are upregulated in response to stress and prevent intracellular proteins from misfolding and aggregating (33). Exogenous administration of HSP70 exerts neuroprotective effects and protects against neurodegeneration in animal models (34). However, under stressful conditions, HSP70 is passively and actively released into the extracellular space triggering inflammation (35). *In vitro* studies have demonstrated that HSP70 and HSP90 stimulate macrophages to release the inflammatory mediators IL-1β, TNF-α -, IL-12, and granulocyte-macrophage colony-stimulating factor. In clinical studies, the relationship between serum Hsp70 and depression is not precise with variable findings between serum levels of HSP70 and depressive symptoms (36). Based on the preclinical data, HSP70 appears to be a promising target for antidepressant therapeutic development.

The unfolded protein response (UPR) plays a prominent role in the etiopathogenesis of depression. The function of the endoplasmic reticulum (ER) is to properly fold proteins and traffic them to various organelles and the cell membrane (37). Stress increases the accumulation of unfolded and misfolded proteins in the ER and in response the UPR signals HSPs, such as Grp94 and GRP78, to refold the proteins. IRE-1 detects when these mechanisms are insufficient to recover protein folding and activates apoptotic and NF-κB signaling pathways leading to the transcription of inflammatory cytokines (38). Timberlake and colleagues found that cortisol levels and GRP78 and GRP94 gene expression were higher in the hippocampus of rats with learned helplessness compared to rats without learned helplessness (39). Moreover, Kim and colleagues found that macrophage cell lines treated with tunicamycin increased the expression of ER stress markers, GRP94 and GRP78, and secretion of IL-6 (40). The preclinical data supports that stress induced activation of the UPR system in the ER is associated with inflammatory cytokines and depressive behaviors. Drug development targeting ER stress has the potential to ameliorate depression. ER membranes express sigma-1 receptors (sig-1R), and preliminary preclinical data suggest that induction of sig-1R alleviates ER stress and represses apoptotic signaling pathways (41). Therapeutics directed at sig-1R are currently under development for a variety of neuropsychiatric disorders.

### 4.3. Gal-3

Gal-3 is a multifunctional protein expressed in peripheral and central immune cells that regulates crucial cellular processes such as growth, proliferation, differentiation, and apoptosis. Gal-3 is released into the extracellular space during the differentiation of monocytes into macrophages (42). Gal-3 activates microglia and innate immune responses, accelerating the secretion of pro-inflammatory cytokines (43). The role of Gal-3 in depression has primarily been studied in patients with comorbid medical conditions. Observational studies in diabetic and obese patients have found that depression is linked to higher levels of gal-3, inflammatory cytokines, and parallel dysregulation of glucose metabolism (44). Moreover, King and colleagues reported that higher levels of Gal-3 were associated with greater depressive symptoms in a community-based sample of patients with heart failure and stroke (45). Emerging literature supports that treatment with hypoglycemic medications, such as metformin, are associated with reduced depressive symptoms and correspondingly lower levels of Gal-3. Moreover, in preclinical studies, glucagon-like peptide-1 agonists, which are utilized in the treatment of type 2 diabetes and obesity, have been shown to reduce Gal-3 and correspondingly ameliorate depression indirectly through neurogenesis (46). Based on the preliminary evidence, Gal-3 is a promising biomarker for monitoring and treating depression in chronic inflammatory states.

## 5. Pattern-recognition receptors

Pattern-recognition receptors (PRRs) are proteins expressed primarily on macrophages, dendritic cells, and microglial cells. Immune cells expressing PRRs detect DAMPs and activate peripheral and neuroinflammation. DAMP-PRR signaling is central to the initiation and perpetuation of the innate immune system. Several PRRs, toll-like receptor-4 (TLR-4) and NLR family pyrin domain containing 3 (NLRP3), have been implicated in depression (47).

### 5.1. NLRP3

NLRP3 is an intracellular PRR that activates downstream signaling cascades leading to activation of inflammasome components. Activated NLRP3 oligomerizes with adaptor proteins and pro-caspase-1 resulting in the formation of the NLRP3 inflammasome (48). The NLR3P inflammasome mediates proteolytic cleave of pro-caspase-1 leading to activation of caspase-1, which cleaves pro-interleukin 1β and 18 into their active cytokine forms (47). IL-1β cytokines have a role in stress responses and depression. IL-1β inhibits hippocampal neurogenesis and contributes to diminishing hedonic responses in susceptible animal models (49). Reversing the effects of IL-1β by means of antagonizing the IL-1R restores hippocampal neurogenesis and reduces depressive-like behaviors (50). Clinical studies have corroborated that depressed patients have higher serum levels of IL-1β compared to non-depressed patients. Moreover, Ferentinos and colleagues reported that depression severity correlated with higher serum levels of IL-1β (51).

In addition, IL-18 has been linked to inflammation and depression. IL-18 binds to the IL-18R and stimulates the production of IFN-γ and pro-inflammatory cytokines. IL-18 has been extensively studied in post-stroke mice with the evidence supporting that IL-18 predicts the risk of developing depression. Moreover, Wu and colleagues found that injecting recombinant mouse IL-18 into the amygdala increased depressive-like behaviors, which was reversed with an IL-18R antagonist (52). The preclinical data strongly support a central role for IL-18 in the development of depression post brain injury. A longitudinal study following post stroke patients reported that plasma levels of IL-18 were independently associated with the development of minor and major depression (53). The depressogenic effects of IL-18 might be secondary to disruption of functional neural networks. Peripheral levels of IL-18 correlate with degree centrality of the left posterior cingulate gyrus and decreased connectivity between the posterior cingulate cortex and the bilateral caudate in depressed patients (54, 55) The imaging data support that IL-18 affects neural networks that modulate motivation and reward processing in depression.

### 5.2. TLR-4

**Figure 3:**
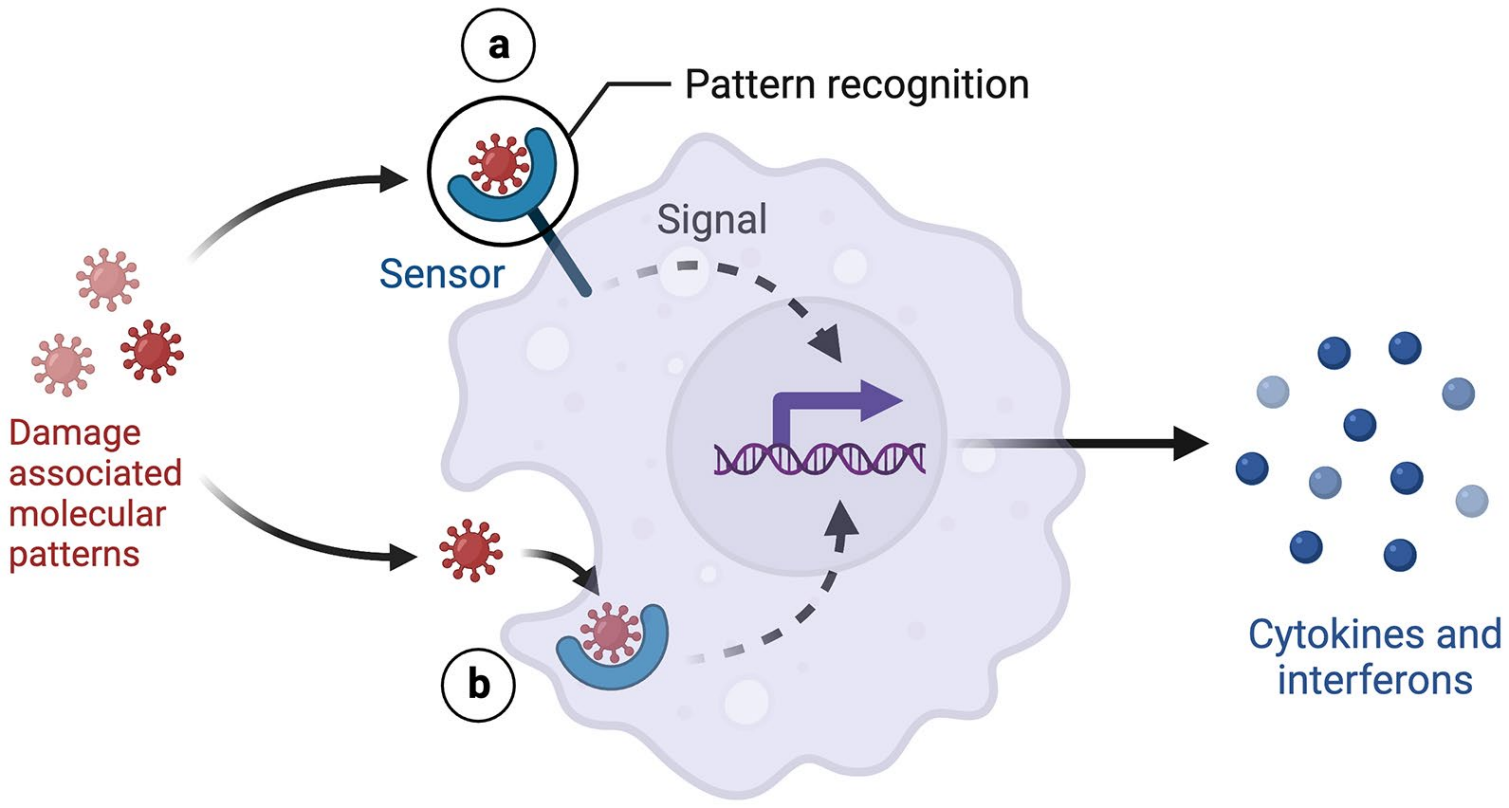
DAMP-PRR signaling. Created with BioRender.com.

TLR-4 is a membrane bound receptor primarily found on macrophages and microglia. Homodimerization of TLR-4 receptors leads to the activation of the enzyme glycogen synthase kinase (GSK-3) and the transcription factor NF-κB, which induce the production of the pro-inflammatory cytokines IL-1β, TNF-α, IL-6, and CXCL10 (56). Repeated restraint or acoustic stress exposure activates the TLR-4 pathway and increases inflammatory cytokines in the prefrontal cortex of mice. Inhibiting TLR-4 prior to restraint or acoustic stress prevents activation of NF-κB and proinflammatory enzymes (57). Similarly, Zhang and colleagues found that chronic social defeat stress increased TLR-4 protein levels in the hippocampus and induced behavioral despair, social avoidance, and anxiety-like behaviors in susceptible mice. Administering fluoxetine reduced the expression of TLR-4 and reversed the depressive phenotype (58). Corroborating the preclinical data, observational studies have reported that depressed patients express higher levels of TLR-4 and NF-κB in peripheral blood mononuclear cells compared to non-depressed patients (59). Additionally, postmortem studies have found that mRNA expression of TLR-3 and TLR-4 is significantly increased in the dorsolateral prefrontal cortex of depressed patients compared to controls (60). These findings support that TLR-4 is a promising treatment target for inflammation in depressed patients (Figure 3).

## 6. Cytokines

Activation of the innate and adaptive immune system has been described in depressed patients. Accumulating evidence implicates cytokines in the pathogenesis of depression particularly in patients with chronic systemic conditions. Cytokines are signaling molecules that regulate the immune system and can either activate or inhibit inflammation. Pro-inflammatory cytokines cross the BBB or are produced by neuronal cells dysregulating neurotransmission and neurocircuitry (61). An imbalance between pro- and anti-inflammatory cytokines represents a distinct pathway for depression. A meta-analysis reported higher levels of serum pro-inflammatory cytokines (IL-1β, IL-2, IL-6, IL-12, TNF-α) and reduced anti-inflammatory cytokines (IL-4, IL-10, transforming growth factor [TGF]-β1) in depressed compared to non-depressed patients (62).

Exogenous IFN-α induced depression provided the earliest support that cytokines contribute to clinical depression. Approximately 30% of patients with hepatitis C develop major depression within the first 3 months of receiving IFN-α (63). Yamano and colleagues reversed INF-α induced stress susceptibility in mice with a corticotropin-releasing hormone receptor antagonist in a dose dependent fashion, suggesting that overactivity of the HPA axis mediates the cytokine depression response (64).

TNF-α is a potent proinflammatory cytokine secreted by activated macrophages directly. TNF-α is critical for activating a host of innate and adaptive immune responses. In particular, TNF-α induces cellular apoptosis and the extracellular release of DAMPs. DAMP-PRR signaling activates macrophages and microglial cells to release interleukins, chemokines, and reactive nitrogen and oxygen species (23). In addition, dendritic cells present DAMP associated antigens to T cell receptors differentiating naïve CD4^+^ T cells into T regulatory cells (Treg) and T helper cells (Th). Treg cells suppress the immune response whereas Th cells trigger immunological responses (65). Elevated serum levels of Th1 and Th17 cells along with a decrease in Treg cells have been reported in depression (66).

Th1 cells release IL-2 and IFN-γ initiating a constellation of inflammatory responses through activation of macrophages, CD8+ T cells, and B lymphocytes. Observational studies have reported an increase in circulating Th1 cells in patients with depression, and effective antidepressant treatment is associated with a reduction in circulating Th1 cells (67). Moreover, Myint and colleagues reported higher ratios of the Th1 cytokine IFN-γ/Il-4 and IFN-γ / TGF-β1 in depressed patients compared to non-depressed patients (68). Therapeutics downregulating IFN-γ have the potential to ameliorate inflammation and depression. Kubera and colleagues reported that monoamine-based antidepressants increased IL-10, a negative immunoregulator of IFN-γ, suggesting that the therapeutic efficacy of antidepressants may be related to their immunoregulatory effects on pro-inflammatory cytokines (69).

Activated macrophages release IL-23, which stimulates the development and differentiation of Th17 cells. Serum levels of IL-17, the signature cytokine of Th17 cells, are higher in depressed patients compared to non-depressed patients, and positively correlate with depressive symptoms (70). IL-17 exacerbates neuronal loss in basal ganglia neuroanatomic circuits important for mood regulation in depression (71). Treatment with a bupropion-SSRI combination reverses dopaminergic neuronal loss and ameliorates depression indicating that IL-17 predicts bupropion-SSRI combination response (72). IL-17 is weakly linked to suicide based on results from the AMAGINE-1 and AMAGINE-2 studies which evaluated the safety and efficacy of Brodalumab, a monoclonal antibody targeting the IL-17RA, for psoriasis. Four patients receiving Brodalumab in the open-label treatment arm completed suicide leading to a black box warning for suicidal ideation and behavior. Schiweck and colleagues postulated that blocking the IL-17RA led to higher circulating IL-17 levels and an increased risk for suicidal behaviors (73). A thorough investigation later revealed there was no causal link between Brodalumab and suicidal behaviors (74). Treatments neutralizing IL-17 have the potential to reduce neurotoxicity and improve depression and suicidality. In animal models, anti-IL-17 treatment rescues depression and anxiety behaviors in mice exposed to cumulative mild stress (75). IL-17 and IL-23 are central neuroinflammatory mediators, and clinical trials are currently underway examining the efficacy and safety of monoclonal antibodies to IL-17 and 23 in treatment resistant depression.

NF-κB is a central pleiotropic mediator of inflammation and induces the expression of multiple cytokines and chemokines. NF-κB has dichotomous effects on inflammation, and depending on the phosphorylation activation site, can either promote or inhibit neuronal growth (76). Wang and colleagues found that chronic unpredictable mild stress increased NF-κB expression and pro-inflammatory cytokines in the frontal cortex and hippocampus of susceptible mice. The depressive-like behaviors were reversed with a TLR4 antagonist suggesting that hyperactive TLR4-NF-κB signaling is involved in the pathogenesis of depression (77). Similarly, Munhoz and colleagues found that chronic unpredictable stress increased LPS activated NF-κB binding in the frontal cortex and hippocampus. NF-κB signaling was blunted with a glucocorticoid receptor antagonist supporting the central proinflammatory role of glucocorticoids in depression (78). In clinical studies, post-endotoxin depressed mood is predicted by higher baseline activity of the NF-*κ*B transcription factor in peripheral blood mononuclear cells (79). Modulation of NF-κB signaling is currently being investigated as an approach to treat major depression (80).

## 7. Blood brain barrier

Proinflammatory cytokine mediated disruption of the BBB is the primary link connecting peripheral and neuroinflammation (81). Under normal conditions, peripheral inflammatory molecules are unable to pass through the blood–brain barrier (BBB). However, during chronic stress and inflammation, the BBB becomes increasingly permeable to immune cells, cytokines, and nitro-oxidative stress molecules. The BBB primarily consists of brain microvascular endothelial cells (BMECs) and astrocytic end feet. BMECs are connected to each other by tight junctions, GAP junctions, and adherens junctions (82). Claudin and occludin are the major transmembrane proteins of tight junctions and are connected to the actin cytoskeleton with zo 1 and zo 2 adaptor proteins. Pro-inflammatory cytokines and astrocytic release of VEGF-A decrease the expression and assembly of claudin and occludin and increase the paracellular permeability of peripheral immune cells (83). Invading macrophages secrete reactive oxygen species (ROS) and matrix metalloproteinases (MMP-1 and MMP-2) further degrading the BBB and perpetuating the cycle of immune cell migration and inflammation. Several pathways to cross the BBB have been described: (1) Entering the brain parenchyma through leaky capillaries in the circumventricular organs and choroid plexus, (2) Direct transportation into the CNS, (3) Binding to vagal afferents and entering the brain, and (4) Direct cellular trafficking (84).

**Figure 4:**
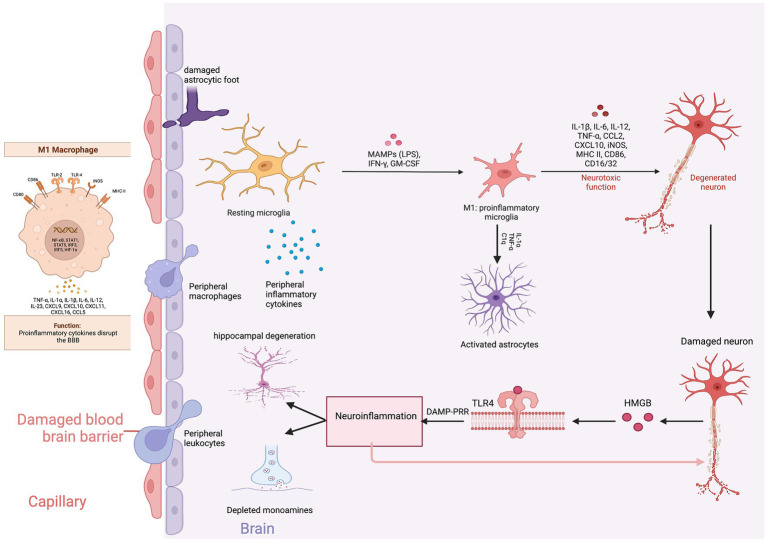
Neuroinflammation and depression. Created with BioRender.com.

The neurobiology of BBB permeability was first elucidated in animal models. Menard and colleagues found reduced expression of claudin-5, an endothelial cell tight junction protein, and abnormal blood vessel morphology in the nucleus accumbens promoted the passage of IL-6 across the BBB. The infiltration of peripheral IL-6 into the brain parenchyma led to the development of depression-like behaviors. Chronic administration of imipramine normalized claudin-5 mRNA expression in the nucleus accumbens and reversed depressive behaviors in stress susceptible mice (81). Similarly, confirmatory studies reported that the expression of claudin-5, occludin, and zo-1 were reduced in the frontal cortex and hippocampus of stress susceptible rats (85). These findings support that claudin-5 is the major gate keeper of the BBB. Treatment with lithium, haloperidol and chlorpromazine significantly increase levels of claudin-5 protein in mice brain microvascular endothelial cells suggesting that psychotropic efficacy might be in part due to improvements in BBB integrity (86) (Figure 4).

## 8. Gliosis

The infiltrating peripheral immune cells release inflammatory cytokines and monocyte chemotactic protein-1 (MCP-1) inducing the migration of macrophages and monocytes into the CNS. Macrophages produce phosphodiesterase-4 (PDE4) leading to the production of pro-inflammatory cytokines (87). The proinflammatory cytokines and chemokines trigger gliosis, a neuro-immune response manifested by the proliferation and hypertrophy of glial cells (astrocytes, microglia, and oligodendrocytes) (88). Mounting evidence supports that gliosis is associated with increased levels of proinflammatory cytokines and reactive oxygen and nitrogen species (RONS) in subcortical limbic brain regions implicated in depression (89).

Depending on the phenotypic expression, microglia can either induce neurotoxicity (M1) or provide neuroprotection (M2). Therefore, the balance between M1 and M2 microglia is critical for maintaining homeostatic synaptic plasticity. During inflammation, microglia polarize toward the M1 phenotype, releasing proinflammatory cytokines and excessive glutamate (90). Translocator protein 18 kDa (TSPO) is expressed on the outer mitochondrial membrane of M1 microglia and is an indirect measure of microglial activation and neuroinflammation (91). PET imaging studies have found an increased uptake of TSPO ligands in the prefrontal cortex, anterior cingulate cortex, and the hippocampal formation in depressed compared to non-depressed patients indicating that microglial activation leads to neuroinflammation (92). Furthermore, a meta-analysis of molecular neuroimaging studies estimated that depressed patients had an 18% increase of TSPO availability compared to non-depressed patients (93). Minocycline, an antibiotic that reduces inflammation through inhibition of microglia, improves depression with effect sizes as high as 0.71 compared to placebo (94). Targeting M1 microglial activation may pave the way for the development of novel therapeutic interventions.

M1 microglia induced astrocytic neurotoxicity is a critical factor contributing to depression. Astrocytes in the hippocampus are crucial for regulating glucocorticoid-mediated feedback to the HPA axis and maintaining cortisol tone. Glial fibrillary acidic protein (GFAP), an astrocytic structural intermediate filament protein, is commonly measured to distinguish astrocytic activity from other glial cells. Mice exposed to chronic daily social defeat expressed reduced GFAP in stress-related brain regions associated with depressive-like behaviors (95). Administering fluoxetine reversed the loss of GFAP-positive astrocytes and ameliorated depressive behaviors (96). Similarly, postmortem studies have confirmed a reduced density of GFAP-immunoreactive astrocytes in brain tissues of patients with a history of MDD or who have completed suicide compared to controls (97). The preliminary data support that astrocytic dysfunction is associated with depression and suicide. Furthermore, a retrospective study found increased GFAP concentrations in the CSF of patients with unipolar depression compared to controls, indicative of astroglia injury (98). Steinacker and colleagues found that serum GFAP levels, at a cut off 130 pg./ml, distinguished MDD from bipolar disorder and schizophrenia with a sensitivity and specificity of 87 and 70%, respectively (99). Therefore, serum GFAP levels might be a useful biomarker candidate to distinguish and monitor the severity of major depressive disorder.

## 9. Oxidative stress

In addition to IL mediated inflammation, an imbalance between free radicals and antioxidants has been implicated in depression. The brain’s high consumption of oxygen makes it particularly vulnerable to oxidative stress compared to other organs and tissues (100). Activated macrophages and microglia release ROS that disrupt cell membranes, cellular DNA, and protein synthesis (101). The high levels of ROS overwhelm the capacity of antioxidant enzymes and disturb redox homeostasis. In addition to the overproduction of ROS, reduced detoxification capacity accelerates cellular damage. Reduced plasma levels of antioxidants (vitamin E, zinc, coenzyme Q10, glutathione) and enzymes (xanthine oxidase and superoxide dismutase) are interrelated with worsening depressive symptoms (102).

Evidence of a pro-oxidative state has been validated in patients with depression. Studies have reported higher levels of oxidative stress byproducts and lower levels of antioxidant enzymes (103). Moreover, correlative studies have found a positive interrelation between oxidative stress by products and pro-inflammatory cytokines suggesting a bidirectional association with the progression of depression. Rybka and colleagues found higher concentrations of malondialdehyde and IL-6 and lower levels of glutathione peroxidase and erythrocyte superoxide dismutase (SOD-1) in depressed compared to non-depressed patients. The authors concluded that the oxidative inflammatory cytokine response represents a molecular pathway underpinning depression (104). Moreover, greater symptoms of depression are positively correlated with higher urinary levels of oxidatively damaged DNA and RNA products (105). Oxidative associated depression is also characterized by IgM-related autoimmune responses directed against neoepitopes from damaged lipids, proteins, and DNA. These autoimmune responses dysregulate key cellular functions and are thought to play a role in the neuroprogression of inflammation and depression (106).

Effective antidepressant treatment reverses the effects of oxidative damage. Khanzode and colleagues reported that SSRIs reduced serum levels of malondialdehyde and increased plasma ascorbic acid levels, which correlated with improvements on the Hamilton Depression Rating Scale (107). Brain Derived Neurotrophic Factor (BDNF) regulates the antioxidant gene transcription factor erythroid 2-related factor 2 (Nrf2) (108). Reduced BDNF inhibits Nrf2 transcription of antioxidant enzymes and increases susceptibility to depressive behaviors in animal models (109). Correspondingly, post-mortem studies have found decreased expression of Nrf2 in the prefrontal cortex of depressed compared to non-depressed patients (110). Administering nrf-2 activators, such as sulforaphane, reduces serum corticosterone, IL-6, and TNF-α, and subsequently, reverses depressive behaviors in mice (111). Therefore, increasing the antioxidant capacity is an effective strategy to reduce inflammation and ameliorate depression. A systematic review reported that diets rich in antioxidant vitamins (A, C, and E) and nutrients (beta carotene, selenium, copper, zinc) reduced the risk of developing depression by about 33% (112).

## 10. Reactive nitrogen species

Nitrosative stress occurs when there is an abnormal increase in the level of nitric oxide (NO) produced by the inducible isoenzymes of NO synthase: neuronal, inducible, and endothelial (113). NO reacts rapidly with superoxide (O_2_^−^) to produce peroxynitrite (ONOO^−^), a potent ER stress oxidant inducing agent. ONOO^−^ inhibits energy metabolism, damages DNA, alters cytoskeletal organization, and impairs cell signal transduction (114). ONOO^−^ nitrates tyrosine residues on proteins, generating 3-nitrotyrosine (3-NT), a biomarker of NO-dependent nitrosative stress. Urine levels of 3-NT are higher in depressed compared to non-depressed patients, implying that nitrosative stress is a major contributor to cytokine induced depression (115).

NO is synthesized from oxygen dependent l-arginine by nNOS in glial cells (116). NO is a second messenger with pleiotropic effects, and at excessive concentrations, overstimulates the NMDA receptor precipitating excitotoxicity (117). Narsapur and colleagues reported that treatment with methylene blue, a NO scavenger, improved depressive symptoms in patients resistant to lithium (118). Similarly, paroxetine inhibits nNOS and subsequently reduces serum levels of NO and improves depression (119). To maintain homeostasis, the enzyme nitric oxide reductase (NOR) inactivates and catalyzes NO to nitrous oxide (N_2_O). Preliminary evidence supports that inhaled N_2_O improves treatment-resistant depressive symptoms (120). Therefore, modulation of NO signaling is a potential therapeutic target for MDD. Nagele and colleagues found that inhaled N_2_O at a concentration of either 25% or 50% significantly reduced scores on the Hamilton Depression Rating Scale (HDRS) at week 2 in patients with treatment resistant depression. The antidepressant effects of N_2_O are mediated through antagonism of the metabotropic glutamatergic NMDA receptors (121). Similarly, preliminary findings in animal models support that inhalation of sub-anesthetic doses of xenon, a potent antiglutamatergic anesthetic, reduces the severity of depressive disorders (122).

nNOS is differentially expressed throughout the brain in patients with MDD. Oliveira and colleagues found increased nNOS CA1 hippocampal immunoreactivity whereas Bernstein and colleagues found reduced nNOS locus coeruleus immunoreactivity in postmortem samples of depressed patients (123). The data suggest that an imbalance in nNOS activity is associated with depression. Preclinical work supports that administration of nNOS inhibitors, such as 7-nitroindole, reduces depressive-like behaviors in obese mice (124). Moreover, Zhou and colleagues found that mice exposed to chronic stress exhibited behavioral despair, which correlated with nNOS overexpression and impaired neurogenesis in the hippocampus. Administering inhibitors of nNOS reversed the effects of chronic stress and increased neurogenesis (125). The data suggest that inhibition of nNOS is a promising novel antidepressant target.

## 11. Neurotransmitters

Pro-inflammatory cytokines reduce the bioavailability of neurotransmitters critical for synaptic communication (61). Pro-inflammatory cytokines decrease monoamine availability through induction of mitogen activated protein kinase (MAPK) signaling, depletion of the cofactor Tetrahydrobiopterin (BH4), and activation of indolamine 2,3 dioxygenase (IDO). Activation of MAPK increases the expression of reuptake pumps for serotonin, norepinephrine, and dopamine, subsequently decreasing the availability of monoamines at the synaptic cleft (126). Inflammatory cytokines also increase the utilization of BH4, a cofactor necessary for nNOS to produce NO. Decreased availability of BH4, a cofactor for the rate limiting enzymes tryptophan hydroxylase and tyrosine hydroxylase, reduces the synthesis of dopamine and serotonin (127). In addition, IDO metabolizes tryptophan, an amino acid precursor to serotonin, thus depleting the availability of serotonin (128).

### 11.1. Quinolinic acid

Activated microglia upregulate IL-18, which increases glycogen synthase kinase (GSK-3β) and IFNγ-dependent IDO expression in glial cells (129). IDO catabolizes tryptophan, a serotonin precursor, to kynurenine. Kynurenine is further converted to either kynurenic acid (KA) or quinolinic acid (QUIN) in astrocytes or microglia, respectively (130). QUIN has neurotoxic effects, and at high concentrations, overstimulates the NMDA receptor resulting in glutamatergic excitotoxicity (131). Moreover, QUIN increases the release and blocks the reuptake of glutamate in astrocytes, thus increasing glutamatergic extracellular concentrations (132). Bay-Richter and colleagues found increased CSF levels of QUIN and decreased levels of KA in suicide attempters. As expected, lower KA levels correlated with severe depressive symptoms and higher IL-6 levels suggesting dysregulation of the kynurenine pathway increases the risk for suicidality (133). QUIN induces neurotoxicity primarily in the striatum, the pallidal formation and the hippocampus (134). This is consistent with morphological and functional findings demonstrating decreased grey matter in the frontotemporal area and reduced connectivity between brain regions linked to reward processing (135).

Meta-analyses have confirmed elevated plasma levels of QUIN in depressed patients compared to non-depressed patients (136). Similarly, post-mortem studies have reported elevated GSK-3β mRNA levels in the hippocampus of patients with major depression. Treatment with GSK-3β inhibitors reduces the production of QUIN and subsequently alleviates depressive, anxious, and aggressive behaviors in animal models (137). Moreover, Verdonk and colleagues reported that decreases in QUIN after a ketamine infusion strongly predicted improvements in MADRS scores in patients with treatment resistant depression (138). The data support that QUIN mediates inflammatory neurotoxicity and disrupts neural circuitry in depression.

### 11.2. Neurotoxicity

Pro-inflammatory cytokines disrupt neurocircuitry implicated in the pathogenesis of depression. fMRI studies have associated higher peripheral levels of IFN-α and TNF- α with increased glutamatergic activity in the basal ganglia and anterior cingulate cortex regions involved in emotion regulation and reward processing (139). Similarly, Capuron and colleagues examined PET changes in patients with HCV treated with IFN-α and found decreased blood-oxygen level dependent responses in the ventral striatal regions during a hedonic gambling reward task. Reduced activity in the ventral striatum was interrelated with reduced motivation, fatigue, and depressed mood on the neurotoxicity scale (140). These findings suggest that cytokines disrupt neurocircuitry likely involved in the pathogenesis of depression.

Neuroinflammatory induced synaptic loss and functional connectivity deficits are considered a final common pathway for depression. The synaptic vesicle glycoprotein 2A (SV2A), a membrane protein expressed primarily in GABAergic containing neurons, is a proxy for estimating synaptic density. Greater symptoms of depression are associated with reduced radioligand binding to SV2A in the dorsolateral prefrontal cortex, anterior cingulate cortex, and hippocampal regions (141). Furthermore, the severity of depression is inversely correlated with neuronal SV2A density and reduced GABAergic inhibitory neurotransmission (142). Garcia-Oscos and colleagues found that administration of IL-6 to cortical rat slice preparations decreased GABAergic inhibition shifting the balance to glutamatergic central hyperexcitability (143). Altered cortical inhibition is implicated in depressive disorders.

Neuronal loss of SV2A results in a global imbalance between glutamatergic excitation and GABAergic inhibition. GABAergic neurons constitute 15 to 25% of the total neuronal population and reduced densities in the prefrontal cortices of mice is associated with major depression (144). Specifically, reduced dendritic inhibition from somatostatin expressing GABAergic interneurons is linked to glutamatergic overexcitability and treatment resistant major depressive disorder. Furthermore, proton magnetic resonance spectroscopy shows reductions of GABA in the occipital cortex, anterior cingulate, and prefrontal cortex in patients with treatment resistant depression (145). Confirmatory post-mortem studies in depressed patients have found reduced somatostatin gene expression in the subgenual anterior cingulate cortex (146). Somatostatin regulates ACTH secretion and loss of somatostatin-expressing interneurons increases glucocorticoid signaling and glutamate neurotransmission in the hippocampus (147). Synchronization of the excitatory to inhibitory (E/I) ratio is important for balancing the signal-to-noise ratio in neural circuits, and reduced inhibition leads to noisier circuits and less efficient information processing (148).

Neuronal circuits are composed of a combination of excitatory and inhibitory neurons. Most neurons are excitatory, and their function is to propagate depolarizing electrical signals to activate downstream neurons. Alternatively, inhibitory neurons hyperpolarize neurons and constrain excitatory neurotransmission (149). Excitatory and inhibitory neurotransmission is critical for regulating the transmission of information between brain areas that control behavior, thought, and emotion. An imbalance between cortical E/I neurotransmission leads to asynchronous and irregular network functioning related to depression. Resting-state functional magnetic resonance imaging has demonstrated reduced functional connectivity between the salience network and right and middle inferior temporal gyrus in depressed patients compared to non-depressed patients. The salience network is important for regulating emotion and cognition, and functional deficits correlate with impairments of cognition and emotion regulation in depression (150). Newer treatments are focusing on restoring the glutamate and GABAergic neurotransmission balance (151). SV2A antidepressant ligand treatments are currently under development to restore the GABAergic tone (152). Similarly, drug discovery is focusing on developing differentiated Kv7 potassium channel openers that generate a hyperpolarizing M-current and reduce hyperexcitability (153) (Table 1).

**Table 1:** Emerging inflammatory biomarkers and treatment targets for depression.

Inflammatory mediators	Inflammatory role	Emerging therapeutics
HPA-axis (16)	*Overproduction of glucocorticoids* elicits immune cells to release pro-inflammatory cytokines	Vasopressin 1 b receptor antagonists Cortisol lowering agents
CRP (6)	CRP is an acute-phase inflammatory protein that activates the complement system *via* C1q	CRP blockers
NF-κB (76)	NFκB controls the transcription of DNA and cytokines and is a central regulator of the *immune system*	NF-κB signaling inhibitors
*IFN*-*γ* (69)	*IFN*-*γ* activates macrophages, natural killer cells, and neutrophils.	*IFN*-*γ* antagonists
TNF-α (154)	TNF-α directly induces cellular apoptosis and the release of DAMPs amplifying the release of proinflammatory cytokines.	Monoclonal antibodies to TNF-α
IL-1β (50)	IL-1β increases the release of TNF-α and IL-6	IL-1β receptor antagonists
IL-2 (67)	IL-2 differentiates Th1 and Th2 effector cells	IL-2 antibodies
IL-6 (11)	IL-6 increases the production of acute phase proteins and neutrophils and differentiates B an T cell proliferation	Anti-IL-6 receptor antibodies
IL-12 (62)	IL-12 promotes T-cell proliferation and increases the production of *IFN-γ* and TNF-α	IL-12 monoclonal antibodies
IL-17 (73)	IL-17 activates astrocytes, microglia, and endothelial cells to release cytokines and chemokines.	IL-17 monoclonal antibodies
IL-23 (70)	IL-23 stimulates the development and differentiation of Th17 cells	IL-23 monoclonal antibodies
PDE-4 (87)	PDE-4 hydrolyzes and inactivates intracellular cAMP, which is a second messenger that suppresses the production of pro-inflammatory cytokines	PDE-4 inhibitors
MMP-1 and MMP-2 (84)	MMP-1 and MMP-2 degrade components of the blood brain barrier	Tissue inhibitors of the metalloproteinases 1–4
MCP-1 (84)	MCP-1 is a chemokine that regulates migration and infiltration of monocytes/macrophages into the CNS	Monoclonal antibody to MCP-1
CXCL10 (56)	CXCL10 promotes monocytes, macrophages, T-cell, and NK cell chemotaxis	Anti-CXCL10 monoclonal antibody therapy
ROS (112)	ROS disrupt cell membranes, cellular DNA, and protein synthesis	Increasing the total antioxidant capacity
RNS (118)	NO is a second messenger with pleiotropic effects, and at excessive concentrations overstimulates the NMDA receptor precipitating excitotoxicity	NO scavengers
nNOS (119)	nNOS synthesizes *NO* from oxygen dependent *l*-*arginine in glial cells*	nNOS inhibitors
TLR-4 (57)	TLR-4 is a PRR that activates the transcription factor NF-κB	TLR-4 antagonists
*Gal-3* (46)	Gal-3 induces *macrophages to produce and release IL-8, IL-6, and TNF-* α	Gal-3 inhibitors
HSP70 (34)	HSP70 helps client proteins fold correctly and are pivotal to maintaining cellular integrity during stress	Exogenous HSP70 administration
NLRP3 inflammasome (48)	NLRP3 mediates proteolytic cleave of procaspase-1 leading to activation of caspase 1, IL-1β and IL-18	Inhibitors of the NLRP3 inflammasome components
GSK-3 β (137)	GSK-3 β promotes IFN-γ dependent IDO enzyme expression	Selective GSK-3 β inhibitors
IDO (131)	IDO catabolizes tryptophan to kynurenine and QUIN, which has potent neurotoxic effects.	IDO enzyme inhibitors
QUIN (138)	Overstimulates the NMDA receptor resulting in glutamatergic excitotoxicity	GSK-3β inhibitors
COX-2 (155)	COX-2 increases PGE-2 resulting in glutamatergic excitability, stimulation of CRF, and the release of pro-inflammatory cytokines	COX-2 inhibitors
Proteoglycans (152)	Reactive astrocytes produce inhibitory factors, such as proteoglycans, which limit the regrowth and formation of new synapses.	Enzymes that cleave the proteoglycan polysaccharide chains.
M1 Microglia (156)	M1 Microglia activate the central innate immune system	Inhibitors of M1 polarization, such as, minocycline EPA, and simvastatin

## 12. Systemic anti-inflammatories

The efficacy and safety of systemic anti-inflammatories has been studied in depressive disorders. Limitations of data interpretation include the small number of studies, moderate to large heterogeneity, variations in diagnostic and treatment planning, and inclusion of patients without evidence of inflammation. Overall, the literature does support that NSAIDs, statins, omega-3 fatty acids, N-acetylcysteine, and COX-2 inhibitors have a small to moderate antidepressant effect sizes over the course of 4 to 12 weeks of treatment. Adjunctive treatment has a larger antidepressant effect size than monotherapy in part due to synergy between monoamine-based treatments and anti-inflammatories. Augmentation with an anti-inflammatory appears to be 52% more effective in reducing symptom severity compared to placebo. Of the anti-inflammatories, NSAIDs, omega-3 fatty acids, and statins, are the most effective at reducing depressive symptoms compared to placebo (157).

### 12.1. NSAIDs

COX-1 and COX-2 enzymes are necessary for the synthesis of prostaglandins involved in inflammation. Prostaglandins increase the release of pro-inflammatory cytokines, suppress Th2 cells, and induce the synthesis of chemokines (158). The COX 2 enzyme is largely induced in the CNS by LPS and proinflammatory cytokines. COX-2 overexpression increases PGE-2 resulting in glutamatergic excitability, stimulation of corticotropin-releasing hormone, and the release of pro-inflammatory cytokines (159). Preclinical studies confirm that chronic unpredictable stress increases the uptake of PET radioligands targeting the COX-2 enzyme and cerebral PGE-2 concentrations in susceptible compared to resilient mice (160).

These findings have led to an increasing amount of research on the potential role of COX-2 inhibitors in the treatment of depression (161). Administering celecoxib, a COX-2 inhibitor, reverses the effects of chronic unpredictable stress and reduces COX-2 expression in a dose-dependent manner (162). A randomized, double-blind, placebo-controlled trial found that the addition of celecoxib to sertraline over 4 weeks improved response and remission rates suggesting a synergistic relationship between celecoxib and sertraline (163). Similarly, Kohler and colleagues found that patients prescribed celecoxib compared to placebo were 7 times more likely to respond to an antidepressant with no increased risks of GI or CV effects (155).

### 12.2. Omega-3 fatty acids

Omega-3 fatty acids, specifically, eicosapentaenoic acid (EPA) and docosahexaenoic acid (DHA), have been studied in depression. EPA and DHA ameliorate inflammation by inhibiting leukocyte chemotaxis, adhesion molecule expression, and the production of inflammatory cytokines and prostaglandins (164). The anti-inflammatory effects are mediated by the production of a distinct class of metabolites known as specialized pro-resolving mediators. Intracranial injection of specialized pro-resolving mediators in mice exposed to chronic unpredictable stress or LPS inhibits astrocyte and microglia activation, reduces the expression of IL-1β and TNF-α in the hippocampus, and reduces the degradation of synapsin (165).

Clinical studies have validated the relationship between omega-3 fatty acids and depression. Chang and colleagues found that depressed patients with hepatitis C treated with IFN- α had lower serum EPA levels and higher scores on neurotoxicity rating scale (NRS) for somatic symptoms compared to non-depressed patients (166). Pretreatment with EPA may reduce the risk of developing depression in patients with hepatitis C treated with INF- α (167). A dose finding study comparing 1 g, 2 g, or 4 g of omega-fatty acid capsules with a 4:1 ratio of EPA:DPA reported that high-doses (4 g/per day) were required for antidepressant efficacy in overweight or obese patients with inflammation (156). Overall, the data indicate that high doses of EPA are required to achieve anti-depressant efficacy.

### 12.3. Statins

Inhibitors of 3-hydroxy-3-methylglutaryl-Coenzyme A (HMG-CoA) reductase commonly known as statins have been studied in depression due to their broad anti-inflammatory effects. Simvastatin treatment in high fat diet or streptozotocin-induced diabetic mice has been shown to rescue depressive-like behaviors, and restore the expression of 5-HT receptors, BDNF, and anti-inflammatory cytokines in the hippocampus (168). Similarly, Na and colleagues reported that administration of atorvastatin to streptozotocin-induced diabetic mice rescued depressive behaviors, attenuated microglial activation in the prefrontal cortex, and reduced the expression of IL-1β, TNF-α, and NF-κB p65 expression in the prefrontal cortex. Overall, the data in animal models supports that statins reduce stress induced depression through amelioration of inflammation (169).

A meta-analysis of RCTs found that the addition of a statin to an antidepressant had a moderate effect on reducing depressive scores compared to placebo at week 8 and 12 with no report of serious adverse events across all trials (170). Statins have varied lipophilic profiles, and the ability to cross the BBB affects antidepressant efficacy and activity. Simvastatin is more lipophilic than other statins, and a study comparing antidepressant outcomes in patients post CABG found that simvastatin had greater antidepressant effects than atorvastatin (171). A large Swedish national cohort study confirmed that statins reduced the odds of depression by 8% compared to placebo, but a subgroup analysis found that only simvastatin significantly reduced the odds of depression (172). Based on the body of evidence, simvastatin appears to be the most promising statin treatment for reducing neuroinflammation in depression.

## 13. Anti-cytokine therapies

A limitation of systemic anti-inflammatories is that they are not specific to cell mediated immunity. To address this limitation, the antidepressant efficacy of targeted monoclonal antibodies to inflammatory cytokines has been studied in randomized clinical trials primarily as a secondary outcome in patients with autoimmune and rheumatological conditions. A meta-analysis of seven randomized controlled trials reported that anti-cytokine treatment had a moderate effect size (Cohen’s d = 0.40) on depression compared to placebo among patients with chronic inflammatory conditions independent of improvements in physical symptoms. Specifically, antibodies against TNF-alpha (Adalimumab, etanercept, infliximab) and IL-6 (tocilizumab) showed statistically significant improvements in depressive symptoms (173). Antibodies to TNF-α are the most studied anti-cytokine therapeutics in depression. A double-blind, placebo-controlled, randomized clinical trial in treatment resistant depression found that infusions of infliximab significantly reduced scores on the HAM-D at week 12 in patients with a baseline CRP concentration greater than 5 mg/L (154). The literature supports that anti-cytokine treatments might be useful as a treatment option for depression associated with an inflammatory component.

Novel pipeline drugs focusing on antibodies to IL-6 and IL-17 show some promise. Sun and colleagues reported that antibodies to IL-6, sirukumab and siltuximab, significantly improved depressive symptoms in patients with rheumatoid arthritis or multicentric Castleman’s disease even after adjusting for disease severity, pain, and physical functioning (174). A study analyzing the efficacy of 3 randomized, double-blind, controlled trials in patients with moderate-to-severe plaque psoriasis, found that ixekizumab, an anti-IL-17 antibody, reduced scores on the QIDS-SR-16 and improved depression at week 12. Moreover, 45% of patients treated with ixekizumab remitted compared to 18% on placebo. The improvement in depressive symptoms correlated with reductions in CRP and the psoriasis area index (175). To date, there is a paucity of published literature on the antidepressant efficacy of monoclonal antibodies to inflammatory cytokines in patients without autoimmune conditions. Additional safety and efficacy studies are needed in treatment resistant patients without a history of autoimmune and rheumatological conditions.

## 14. Transforming growth factor beta (TGF- β)

TGF-β is a multifunctional set of pleiotropic cytokines that control cell proliferation, differentiation, and motility of neural cells. Canonical TGF-β signaling exerts anti-inflammatory neuroprotection and influences memory formation and synaptic plasticity (176). TGF-β exists in three isoforms, of which TGFB_1_ is the most widely studied in depression. TGFβ_1_ binds to the TGF beta receptor 1 (TGFBR1) on microglial cells and inhibits the production and release of cytokines, chemokines, adhesion molecules, and ROS through activation of p38 MAPK and Akt phosphorylation (176). Silencing the TGFBR1 in microglia abolishes the neuroprotective and anti-inflammatory properties of TGF-β1 in animal models. Moreover, a study found that inhibiting p38 MAPK prevented TGF-β1 from inhibiting microglial activation and 1-methyl-4-phenylpyridinium (MPP^+^)-induced dopaminergic neuronal toxicity. The preclinical data suggest that impaired TGFβ_1_ signaling increases microglial activation and inflammation in depression (177). Moreover, a growing body of research suggests that the antidepressant effects underlying r-ketamine are dependent on microglial TGFβ_1_. Zhang and colleagues reported that administering neutralizing antibodies to TGF-β1 blocked the antidepressant effects of (*R*)-ketamine in chronic social defeat stress mouse models (178). Further supporting the immunoregulatory role of TGFβ, pre-clinical research has found that inhalation of recombinant TGF-β1 elicits neurogenesis and reduces depressive behaviors in chronic social defeat stress (CSDS) models (179).

More recent developments have focused on understanding the clinical application of TGFβ_1_ in depression. Studies have not shown a consistent relationship between plasma levels of TGFβ_1_ and depression, nonetheless, TGFβ_1_ appears to regulate inflammatory processes underlying MDD. Preliminary evidence supports that childhood maltreatment mediates a higher concentration of TGFβ_1_ in adults with major depressive disorder suggesting an epigenetic role for TGFβ_1_ in stress related disorders (180). Caraci and colleagues reported that TGF-β1 plasma levels were reduced in patients with major depression and correlated with depression severity and treatment resistance. Administering sertraline and venlafaxine increased serum levels of TGF-β1 and reduced depression (181). In contrast, despite a response, Rush and colleagues found that ECT did not normalize reduced levels of TGF-β in patients with melancholic depression (182). Larger and well-designed studies are needed to determine the prognostic and therapeutic potential of TGF-B1 in depression.

## 15. Neurotrophic factors

Neurotrophic factors are key molecules that play important functions in the regulation of immune cell activation and neuro-survival. The primary neurotrophic factors (BDNF, NGF, and NT) reduce neuroinflammation through activation of specific tyrosine kinase receptors (TrkA TrkB, TrkC) and downstream signaling of the PKC pathway, Ras/MAPK pathway, and PI3 pathway resulting in neuronal remodeling and formation of novel synapses (183). The activation of these multiple signaling cascades results in neuronal remodeling and adaptive circuitry functioning countering inflammation mediated neurotoxicity (184).

Neurotrophic factors and downstream signaling effects play a critical role in synaptogenesis and plasticity. Therefore, modulating neurotrophic cascade signaling is an effective strategy to ameliorate symptoms of depression rapidly and effectively. As such, innovative treatments to boost neurotrophic factors have been developed to counteract stress and inflammation. To date, drug discovery has primarily focused on indirectly boosting endogenous neurotrophic factors by antagonizing N-methyl-D-aspartate (NMDA) or potentiating alpha-amino-3-hydroxy-5-methyl-4-isoxazole propionic acid (AMPA) (185, 186).

Direct administration of exogenous neurotrophic factors has been limited by pharmacokinetic factors as well as safety and efficacy concerns. Exogenous administration of neurotrophic factors is limited by blood–brain-barrier (BBB) permeability, poor half-life, and rapid degradation (183). Moreover, the preliminary data has been inconsistent as neurotrophic factors exert multiple pleiotropic off-target effects potentially interfering with restoration of synaptogenesis. To overcome these limitations, low molecular weight dimeric dipeptides that mimic BDNF loop 4 have been designed to specifically activate the tyrosine kinase B receptor. Two BDNF mimickers currently under investigation are dipeptide *N*-monosuccinyl-L-seryl-L-lysine and 7,8-Dihydroxyflavone (187). Novel therapeutics specific to the neurotrophic downstream signaling cascade effects are currently under development for depression (Table 2).

**Table 2:** Novel anti-inflammatory drug candidates for depression.

Anti-Inflammatory Mediators	Anti-inflammatory Role	Emerging therapeutics
TGF-β1 (179)	TGF-β1 promotes neurogenesis and plasticity	Intranasal administration of recombinant TGF-β1
Superoxide dismutase (104)	Superoxide dismutase is an antioxidant enzyme that catalyzes the dismutation of superoxide radicals to either molecular oxygen or hydrogen peroxide.	Antioxidant enzyme mimetics
Nrf2–ARE signaling (111)	Nrf2–ARE signaling regulates the expression of genes that encode for proteins involved in antioxidant defense and detoxification.	Nrf2 activators
PI3K-AKT pathway (13)	Regulates inflammation by upregulating key anti-inflammatory cytokines (IL-10) and inhibiting proinflammatory cytokines	Flavanone modification of PI3K-AKT signaling
SV2A (152)	SV2A is a membrane bound protein that induces GABA neurotransmitter release and restores the E/I imbalance	SV2A receptor ligands
K_v_7.1–K_v_7.5 Potassium Channels (153)	K_v_7.1–K_v_7.5 channels hyperpolarize neural cells, reduce excitation, and maintain resting membrane homeostasis	Kv7.2–7.5 potassium channel agonists.
Claudin-5 (86)	Claudin-5 is a critical tight junction protein in the blood brain barrier	Antidepressants that restore claudin-5 expression.
Pro-resolving mediators (165)	Pro-resolving mediators are enzymatically derived from essential fatty acids and serve as a novel class of immunoresolvents.	EPA supplementation

## 16. Conclusion

Inflammation mediates the relationship between chronic stress and the evolution and progression of depression and comorbid conditions. Persistent stress creates a disruptive cycle between allostatic loading, dysregulated glucocorticoid functioning, overactivation of the immune system, and the excessive release of inflammatory molecules, tilting the balance toward a pro-inflammatory state. The extracellular release of DAMPs and DAMP-PRR signaling sets in motion a self-perpetuating cycle of inflammation. The perpetual flux of inflammatory mediators disrupts the blood–brain barrier allowing for cellular and cytokine trafficking into the CNS and activation of glial cells. Glial cell cross talk with peripheral immune cells and cytokines is vital for orchestrating a bi-directional inflammatory network between the periphery and central nervous system. The cascade of pro-inflammatory cytokines and oxidative stress induces neurotoxicity, perturbs neurotransmitter functioning, and globally disrupts the E/I balance within neural circuits related to depression. Reduced gray matter volumes in frontolimbic brain structures and aberrant functional connectivity in reward pathways underlie the biological basis of neuroinflammatory depression. Current monoamine-based treatments reduce neuroinflammation through multiple mechanisms however their effects are limited by delayed therapeutic responses. The diversity of cell mediated immunities, cytokines, and nitro-oxidative stress contributing to depression provides an opportunity to discover stable diagnostic and prognostic biomarkers for neuroinflammatory depressive phenotypes. The relationship between neuroinflammation and depression sets the stage for the development of novel pharmaceuticals. Randomized controlled trials have predominantly focused on the efficacy of systemic anti-inflammatories with preliminary findings demonstrating improvements in depressive symptoms. Systemic anti-inflammatories appear to be promising, however, tailoring treatments to the specific neurobiological mechanism has the potential to robustly improve antidepressant outcomes. Targeted therapies have been limited to animal models or patients with concomitant autoimmune and rheumatological conditions. Future clinical trials should focus on testing therapeutics that regulate the HPA axis, modulate immune cells responses, antagonize inflammatory cytokines, reduce nitro-oxidative stress, and mimic neurotrophic factors with the goal of restoring the E/I imbalance and neural circuitry functioning.

## Author contributions

SH prepared and supervised the draft, contributed to the article, and approved the submitted version.

## Conflict of interest

The author declares that the research was conducted in the absence of any commercial or financial relationships that could be construed as a potential conflict of interest.

## Publisher’s note

All claims expressed in this article are solely those of the authors and do not necessarily represent those of their affiliated organizations, or those of the publisher, the editors and the reviewers. Any product that may be evaluated in this article, or claim that may be made by its manufacturer, is not guaranteed or endorsed by the publisher.
